# A Gas Pressure Scale Based on Primary Standard Piston Gauges

**DOI:** 10.6028/jres.115.027

**Published:** 2010-12-01

**Authors:** Douglas A. Olson, R. Greg Driver, Walter J. Bowers

**Affiliations:** National Institute of Standards and Technology, Gaithersburg, MD 20899

**Keywords:** gas pressure scale, piston gauge, primary standard piston gauge, pressure measurement, secondary standard piston gauge

## Abstract

The National Institute of Standards and Technology (NIST) has redefined its gas pressure scale, up to 17 MPa, based on two primary standard piston gauges. The primary standard piston gauges are 35.8 mm in diameter and operate from 20 kPa to 1 MPa. Ten secondary standard piston gauges, two each of five series of the Ruska 2465 type, with successively smaller diameters form the scale extending up to 17 MPa. Six of the piston gauges were directly compared to the primary standards to determine their effective area and expanded (*k* = 2) uncertainty. Two piston gauges operating to 7 MPa were compared to the 1.4 MPa gauges, and two piston gauges operating to 17 MPa were compared to the 7 MPa gauges. Distortion in the 7 MPa piston gauges was determined by comparing those gauges to a DH Instruments PG7601 type piston gauge, whose distortion was calculated using elasticity theory. The relative standard uncertainties achieved by the primary standards range from 3.0 × 10^−6^ to 3.2 × 10^−6^. The relative standard uncertainty of the secondary standards is as low as 4.2 × 10^−6^ at 300 kPa. The effective areas and uncertainties were validated by comparison to standards of other National Metrology Institutes (NMIs). Results show agreement in all cases to better than the expanded (*k* = 2) uncertainty of the difference between NIST and the other NMIs, and in most cases to better than the standard (*k* = 1) uncertainty of the difference.

## 1. Introduction

Gas operated piston gauges (also known as pressure balances) are used as primary pressure standards throughout the world [1–3], with relative standard uncertainties approaching 3 × 10^−6^. A piston gauge is a round piston fitted in a matching cylinder; the piston is loaded with weights of known mass and density. The piston is marginally smaller than the cylinder, and fluid fills the gap between the two components. Fluid pressure acting over the area of the piston, plus fluid shear stresses acting on the walls of the piston, balances the gravitational force of the weights^1^. By changing the amount of mass loaded on the piston, a single piston gauge can generate a range of pressures. The characterization of a piston gauge as a pressure standard involves determining the *piston gauge effective area*, *A_p_*, such that when the forces are divided by *A_p_* the result is the fluid pressure, *p*, beneath the piston. Or,

(1) $$p=\frac{\sum_{i}m_{i}g\left(1-\frac{\rho_{a}}{\rho_{mi}}\right)}{A_{p}}.$$

*m_i_* and *ρ_mi_* are the masses and densities of the individual weights, *g* is the acceleration due to gravity, and *ρ_a_* is the density of the air surrounding the weights. The effective area is dependent on temperature through thermal expansion of both the piston and cylinder; this effect is factored out to give the *constant temperature effective area A_e_*:

(2) $$A_{p}=A_{e}\left(1+\left(\alpha_{p}+\alpha_{c}\right)\left(T-T_{r}\right)\right).$$

*A_e_* is defined for the reference temperature, *T_r_* (taken at NIST as 23 °C), *α_p_* and *α_c_* are coefficients of thermal expansion for the piston and cylinder, and *T* is the temperature of the piston gauge.

Establishing piston gauges as primary pressure standards at uncertainties comparable to mercury manometers has been the result of improvements in dimensional metrology, the ability to manufacture straight and round large piston/cylinder assemblies of at least 35 mm in diameter, and the application of modeling techniques to calculate the effective area of the piston gauge from the dimensional data. A piston gauge is considered a primary pressure standard if its characterization does not depend on comparison to another pressure standard. NIST has two primary standard piston gauges of 35.8 mm nominal diameter that operate up to 1 MPa. In order to achieve higher pressures, successively smaller diameter piston gauges are required so that the amount of mass loaded on the piston gauge is not excessive.

Although smaller diameter piston gauges can in principle be characterized as primary standards using the same methods as for large diameter gauges, this is not done for a number of practical reasons. Uncertainties in dimensional measurements of diameters in round artifacts are roughly independent of size. The relative uncertainty in effective area (or pressure) based on dimensional metrology therefore varies inversely with the diameter. Dimensional measurement of the internal diameters of cylinders becomes difficult for diameters less than 3 mm. Piston and cylinders distort as the pressure is increased from ambient; above 1 to 2 MPa such distortion becomes significant when compared to the dimensional uncertainty obtained at ambient pressure. At present there are no accepted methods for measuring piston and cylinder diameters at elevated pressures.

At NIST, piston gauges of diameters smaller than 20 mm are characterized by comparison to other piston gauges, rather than as primary standards. Those piston gauges which are *calibrated* in this manner are referred to as secondary standards. The collection of primary and secondary standards forms a gas pressure scale which transfers the SI unit of pressure to NIST customers, at pressures^2^ up to 17 MPa. This paper describes the methods for characterizing the primary and secondary standard piston gauges, the uncertainties of the piston gauges, and the validation of those uncertainties through comparisons to other pressure standards at NIST and other National Metrology Institutes (NMIs).

## 2. The Old and New Gas Pressure Scales

The gas pressure scale which existed prior to 2008 used secondary standard piston gauges traceable to a mercury manometer known at NIST as the Gas Thermometer Manometer (GTM) [4]. A single piston gauge, designated at NIST as PG28, was calibrated against the GTM at 27 kPa and 95 kPa [5] in absolute mode.^3^ PG28 is a Ruska^4^ 2465 model piston gauge, with a 20.7 mm diameter solid piston that is used up to 300 kPa. To extend the range of the pressure scale to higher pressures, 10.3 mm diameter piston gauges were calibrated against PG28 over the overlapping portion of the pressure range, and then 3.3 mm diameter piston gauges were calibrated against the 10.3 mm diameter piston gauges. Distortion in effective area was neglected for all piston gauges operating at 7 MPa and less. The GTM was decommissioned in the early 1990s, after which PG28 was checked for stability against the NIST ultrasonic interferometer manometer (UIM). Relative standard uncertainties of the secondary standard piston gauges used for customer calibrations ranged from 6.5 × 10^−6^ to 20 × 10^−6^.

**Fig. 1:**
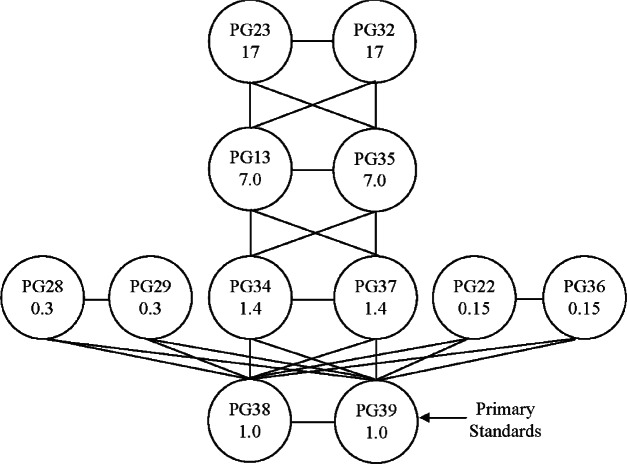
NIST pressure scale for gas primary and transfer standard piston gauges. Circles represent piston gauge standards; the number in a circle is maximum pressure in MPa. Lines between circles represent comparisons between piston gauges.

The new gas pressure scale is shown schematically in Fig. 1. The circles represent the piston gauges in the scale, and the lines represent the calibrations or comparisons between the gauges. The scale is based on two primary standard piston gauges, PG38 and PG39, that operate up to 1 MPa. All of the secondary standard gauges are traceable to the primary standard gauges. Three sets of twin Ruska 2465 piston gauges are calibrated directly against PG38 and PG39: two in the TL series (PG22 and PG36), two in the TTL series (PG28 and PG29), and two in the C series (PG34 and PG37). Ruska 2465 V series gauges (PG13 and PG35) are calibrated against the C series gauges, and Ruska 2465 D series gauges (PG23 and PG32) are calibrated against the V series gauges. Gauges within the series are compared to each other. Characteristics of the piston gauges which form the scale are summarized in Table 1. Details of the determination of the effective area follow in Secs. 2 and 3.

The new pressure scale offers two major advantages over the old scale which contribute to the reduction in uncertainties that are described in the following sections. The first is that PG38 and PG39 operate to pressures a factor of 10 higher than the GTM and a factor of 3 higher than the UIM. This is sufficient to characterize the distortion for the TL, TTL, and C series gauges. The second advantage is that piston gauges are much easier to use than manometers. Hence it is practical to calibrate many of the secondary standard piston gauges against PG38 and PG39 and to re-check the piston gauges for stability at regular intervals.

## 3. Primary Standard Gas Piston Gauges

The primary standard piston gauges were acquired from Ruska in 1989. More extensive details on the method for characterizing them can be found in [2] and [6]. They operate in both absolute and gauge mode, from 20 kPa to 1 MPa.

**Fig. 2:**
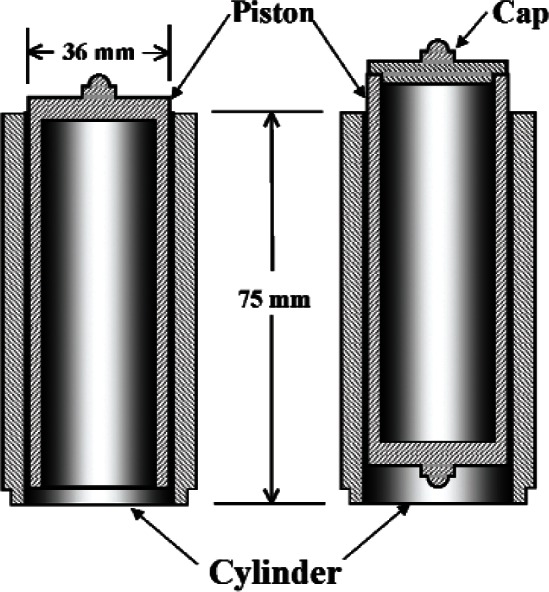
Schematic diagram of the PG38 and PG39 piston/cylinder assembly with the piston in upright (left) and inverted (right) orientations. The cap on the right is used to support the weight carrier plus mass elements.

**Fig. 3:**
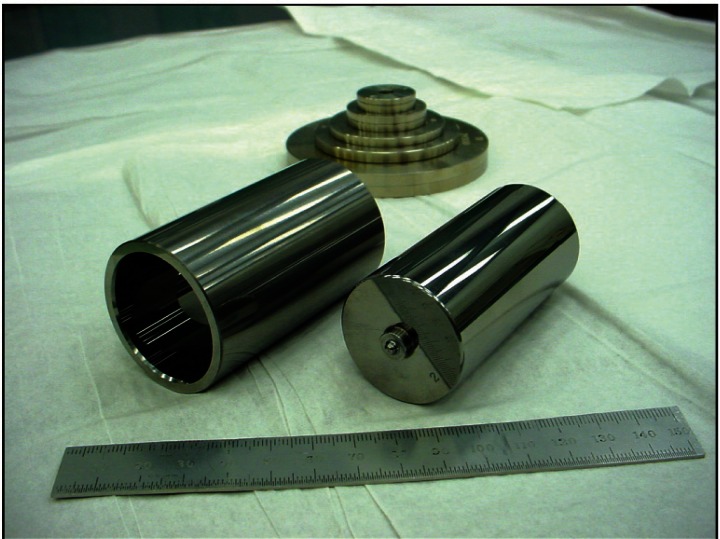
Picture of PG39 cylinder (left), piston (right), and mass set (top). Closed end of piston is shown.

A cross-section of the piston/cylinder assemblies is shown in Fig. 2, and a picture of the assemblies is shown in Fig. 3. The assemblies are “twins” in the sense that they were made from the same casting of tungsten carbide and have the same nominal dimensions. The pistons are hollow, with the hollow end pointed downward in normal operation as shown in Fig. 1 on the left-hand side. Their nominal diameters are approximately 35.8 mm and their length is 75 mm. The radial clearance between pistons and cylinders is about 600 nm. The construction of the pistons is such that they can be inserted into their cylinders either upright or inverted. When operated in the inverted configuration, a special cap with a spherical pivot is placed onto the hollow end to allow the loading of masses. That cap is not sealed to the piston. In the upright position, the interior of the piston is subjected to the system pressure, whereas in the inverted position the piston interior is subjected to ambient pressure. The two orientations of the piston have a different calculable value for the pressure coefficient (relative change in *A_e_* with pressure). The constant temperature effective area (henceforth referred to as effective area) of both PG38 and PG39 is given by the linear distortion equation:

(3) $$A_{e}=A_{0}(1+b_{1}p).$$

Here, *A*_0_ is the effective area at atmospheric pressure and the reference temperature, 23 °C, and *b*_1_ is the pressure distortion coefficient.

There are two components in the establishment of PG38 and PG39 as primary standards. The first is the dimensional measurements of the piston and cylinder diameters; the second is the analysis of that data with force and distortion models to determine the effective area. The modeling and dimensional measurements are used to determine both *A*_0_ and the *b*_1_. The results are verified by comparisons of the effective area of PG38 and PG39 relative to each other when operated as pressure standards. They are further validated by comparison of the gauges to the NIST mercury manometer, which is an independent primary pressure standard traceable to the density and speed of sound of mercury.

In 1999, PG39 was dimensioned by Physikalisch Technische Bundesanstalt (PTB) using a state-of-the art diameter and form comparator in which a calibrating laser interferometer is integral to the apparatus. Absolute diameters were measured at four places on the piston and four places on the cylinder, with a standard uncertainty of 15 nm. Relative roundness was measured at 5 latitudes and relative straightness was measured at 8 longitudes. In 2003, both PG38 and PG39 were measured at PTB with the same device as PG39 was measured in 1999. This time, absolute diameters were measured at 10 places on the piston and 10 places on the cylinder. Four of the locations on PG39 were the same in 2003 as in 1999; the relative difference from 1999 to 2003 ranged from − 0.1 × 10^−6^ to − 0.8 × 10^−6^. The standard uncertainty of the absolute diameters in 2003 was 12.5 nm and 25 nm for the piston and cylinder, respectively. Relative roundness and relative straightness were measured again in 2003 for both pistons and both cylinders, at 5 latitudes for roundness and 8 longitudes for straightness. The standard uncertainty for the roundness and straightness measurements was 50 nm. The 2003 data for both PG38 and PG39 showed that the pistons were round to within the standard uncertainty of measurement. Changes in diameter with height for both pistons and both cylinders were larger than the standard uncertainty of the measurement.

If the uncertainty of the effective area were based on the uncertainty of the dimensional measurements only, they would imply that *A*_0_ has a relative standard uncertainty of 1.0 × 10^−6^. However, the low uncertainty of the dimensional measurements requires that we consider the appropriate model for converting the measurements into “effective area” when the piston gauge is used for generating pressure. The model needs to account for all of the forces on the piston: the external mass load, the normal pressure force on the piston base, the shear forces on the piston flanks, and the normal forces on the piston flanks. It also needs to account for the complete dimensional data which describes the artifacts. In the analysis which establishes PG38 and PG39 as primary standards [2], the data from PTB on roundness, straightness, and absolute diameters were reconstructed in the form of cylindrical “bird cages” providing longitudinal and latitudinal crevice (piston-cylinder gap) variation. Forces were computed assuming two models of nitrogen gas flow behavior in the crevice: (1) viscous flow, and (2) flow of gas that interpolated between molecular flow and viscous flow. The effect of operating mode (gauge or absolute) was evaluated for both models. A complete mathematical description of the models is given in [6].

The results of the two flow models, including the dimensional uncertainty, gives a distribution of *A*_0_ values. The accepted value for *A*_0_ was taken as the average of the maximum and minimum value of the results, and the standard uncertainty, *u*(*A*_0_), as one half of the difference between the maximum and minimum.^5^ Statistically this means that the distribution of *A*_0_ from the models is part of a normal distribution, and that the maximum and minimum results represent about a 2 out of 3 chance that the true quantity lies between those values. The relative standard uncertainty in *A*_0_ evaluated in this way is 3.0 × 10^−6^ for both PG38 and PG39. The largest *A*_0_ occurred for the viscous flow model, and the smallest *A*_0_ occurred for the interpolated flow model in absolute mode.

The value for the pressure distortion coefficient, *b*_1_, was determined from elasticity theory. Two model implementations of elasticity theory were considered. In one, both the cylinder and piston were modeled as infinitely long components subjected to radial forces due to the pressure on the walls, which allowed using analytical formulae. These formulae require a constant pressure (*p*_g_) in the piston-cylinder gap, even though the gap pressure varied from *p* at the gap entrance to ambient at the top. The formulae were solved for *p*_g_ = 0, *p*/2, and *p*. In a second model, finite-element analysis was used to include the constraint of the closed-end of the piston (Fig. 2) and vertical loading on both the piston and cylinder; these constraints produce two-dimensional stresses. The two models and three gap boundary conditions produced a distribution of *b*_1_ values from 7.95 × 10^−12^ Pa^−1^ to 10.0 × 10^−12^ Pa^−1^. The accepted value, 8.97 × 10^−12^ Pa^−1^, was chosen as the average of the maximum and minimum, and the standard uncertainty was taken as one-half the differ ence. To within the standard uncertainty of the distortion models, both PG38 and PG39 have the same *b*_1_ and uncertainty in *b*_1_ due to modeling. The combined standard uncertainty *u*(*b*_1_) includes the standard uncertainty in the Young’s modulus added in quadrature. *u*(*b*_1_) equals 1.12 × 10^−12^ Pa^−1^.

NIST also realizes pressure with a primary standard mercury manometer known as the ultrasonic interferometer manometer (UIM) for pressures up to 360 kPa [7–10]. PG38 and PG39 have been compared numerous times since 1989 to the UIM, serving as check standards to confirm UIM stability and also to confirm the stability of the piston gauges. The combined relative standard uncertainty of the UIM from 20 kPa to 360 kPa is 2.6 × 10^−6^. All comparisons of the UIM to the piston gauges have shown agreement to within one standard deviation of the combined standard uncertainty of the difference, with UIM pressures both higher and lower than those of the piston gauges. These comparisons show the combined stability of the UIM and the piston gauges, and given the independent nature of the realization technique, the likely stability of each method.

**Fig. 4:**
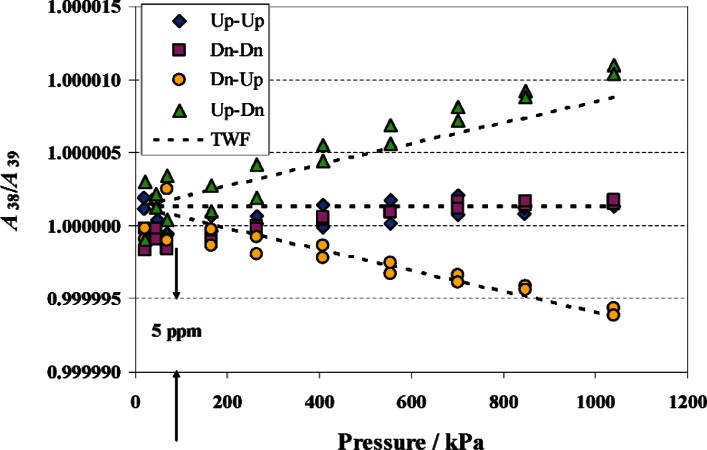
The ratio of the effective area for PG38 to that of PG39 (*A*_38_/*A*_39_). Symbols indicate ratios from crossfloat measurements of PG38 versus PG39 for different combinations of piston orientation (Up-Dn means PG38 upright, PG39 inverted). The dashed lines indicate ratios based on thick wall formulae (TWF) from elasticity theory and *A*_0_ from dimensional characterization.

PG38 and PG39 have been compared directly to each other from 20 kPa to 1 MPa, utilizing the unique feature mentioned earlier that both can be operated in the upright and inverted positions. This comparison measures the area ratio *A*_38_/*A*_39_. Four comparisons (PG38-up, PG39-up; PG38-down, PG39-down; PG38-up, PG39-down; PG38-down, PG39-up) were performed, which were compared to predictions from the distortion models. With both gauges in the same orientation, the distortion coefficients should be the same and the slope (Δ*b*_1_) of *A*_38_/*A*_39_ should be zero. With the two opposing orientations, the models predict (Δ*b*_1_) = ± 7.2 × 10^−12^ Pa^−1^. Figure 4 shows the results of the four comparisons along with the predicted slopes from the analytical models. There is good agreement between the experimental result and the modeling. This helps confirm the use of elasticity theory to establish *b*_1_ and its uncertainty.

## 4. Secondary Standard Gas Piston Gauges

The secondary standard piston gauges are all Ruska 2465 gas piston gauges that NIST acquired from 1972 to 1988. These piston gauges were part of the old pressure scale and the majority of them were also used as transfer standards in the NIST calibration service. The NIST piston gauge numbering system is chronological; PG13 is older than PG22 which is older than PG28, etc. Each gauge is defined by its effective area, given in the form of Eq. (3). The characterization of the gauge is the process of determining the coefficients *A*_0_ and *b*_1_, and the uncertainty of the effective area, *u*(*A_e_*). In some cases, *b*_1_ may be set to zero. Characteristics of the secondary standard piston gauges are summarized in Table 1.

The general procedure for characterization is the same for all the secondary standards: each gauge of a series is calibrated against two gauges with lower uncertainty (lower circles on the chart, Fig. 1); then, the two gauges within a series are checked for consistency by a direct experimental comparison to each other. The data from the calibrations to the lower uncertainty standards is fit as a common set, and linear regression is used to determine coefficients *A*_0_ and *b*_1_. As an example, PG28 and PG29 are both calibrated against PG38 and PG39 and then the data is fit to eq. (3). Their ratio of areas, PG28 to PG29, is then found both by the ratio of calibration equations and by direct experimental comparison.

This method is sufficient for gauges of the TL, TTL, and C series due to the overlap between the pressure ranges of the primary standards and the secondary standards. In characterizing the V series, overlap between the maximum pressure of the C series (1.4 MPa) and the V series (7 MPa) is insufficient to determine distortion coefficient *b*_1_. In that case, elasticity theory is used to calculate the distortion of an auxiliary 7 MPa piston gauge of simpler design, which is then calibrated against a V series gauge to determine *b*_1_. The D series gauges are calibrated against the V series gauges.

The calibration of one piston gauge against another piston gauge requires establishing equal pressures between the gauges. This is done by connecting a fluid line between the bottoms of the pistons. A piston gauge is a pressure generator, so if the pressure produced by each is not equal, the mass on one or both of the gauges is adjusted. The pressure from each piston gauge is given by (combining Eqs. (1) and (2)):

(4) $$p=\frac{\sum_{i}m_{i}g\left(1-\frac{\rho_{a}}{\rho_{mi}}\right)}{A_{e}\left(1+\left(\alpha_{p}+\alpha_{c}\right)\left(T-T_{r}\right)\right)}.$$

The numerator is the sum of the forces from the masses (adjusted for ambient buoyancy), and the denominator is the effective area at the operating temperature of the gauge. Equating the pressure from the two gauges (with a correction for the difference in elevation), and using the subscript nomenclature of *R* for the previously calibrated *Reference* gauge and *T* for the unknown *Test* gauge, the effective area of the *Test* gauge *A_e_*,*_T_* at 23 °C is given by:

(5) $$\begin{array}{l} A_{e,T}=A_{e}{,}_{R}\cdot \\ \frac{\sum m_{i,T}\left(1-\frac{\rho_{a}}{\rho_{mi,T}}\right)}{\sum m_{i,R}\left(1-\frac{\rho_{a}}{\rho_{mi,R}}\right)}\cdot \frac{\left(1+\left(\alpha_{p,R}+\alpha_{c,R}\right)\left(T_{R}-23\right)\right)}{\left(1+\left(\alpha_{p,T}+\alpha_{c,T}\right)\left(T_{T}-23\right)\right)}\cdot \\ \cdot \left(1+\frac{\left(\rho_{f}-\rho_{a}\right)gh}{p_{T}}\right). \end{array}$$

*ρ_f_* is the density of the fluid at pressure *p*, and *h* is the difference in reference levels of the gauges. If the gauges are operated in absolute mode, then *ρ_a_* is zero. Eq. (5) is the measurement equation for the test gauge effective area. Through the calibration, *A_e_*,*_T_* is determined over a range of pressures.

The measured data from Eq. (5) is fit to Eq. (3) using a least squares method that minimizes the squares of the residuals of the fitted effective area from the measured effective area at the data points.

The uncertainty of the effective area of the test gauge is determined using methods described in [11]. Type A uncertainties are evaluated from the statistics of the fit. The Type B uncertainty arises predominantly from the method of propagation of uncertainty as applied to Eq. (5). In some cases, there are other Type B sources of uncertainty that must be considered.

### 4.1 Characterization of TL, TTL, and C Series Secondary Standards

The characterization of these six gauges is similar in that they are all directly calibrated against primary standards PG38 and PG39. The calibrations are performed in gauge mode, as there is currently no provision for enclosing and evacuating the space surrounding PG38 and PG39. The TL and TTL gauges are both 20.3 mm nominal diameter. TTL gauges PG28 and PG29 consist of solid tungsten carbide pistons and tungsten carbide cylinders, whereas TL gauges PG22 and PG36 use hollow stainless steel pistons and tungsten carbide cylinders. The hollow stainless steel piston has less mass, allowing the TL gauges to operate to a lower pressure. The C series piston gauges (PG34 and PG37) operate to 1.4 MPa and are made of tungsten carbide.

**Fig. 5:**
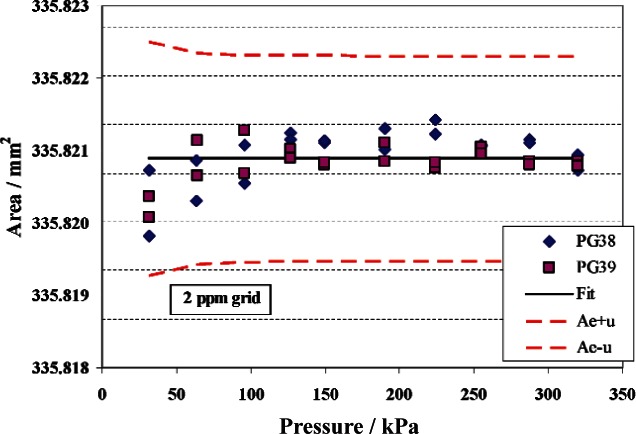
Effective area of PG28. Symbols are measured data from PG38 and PG39, solid line is fit of data with *b*_1_ constrained to zero, and dashed lines are the fitted area plus or minus the standard uncertainty.

**Fig. 6:**
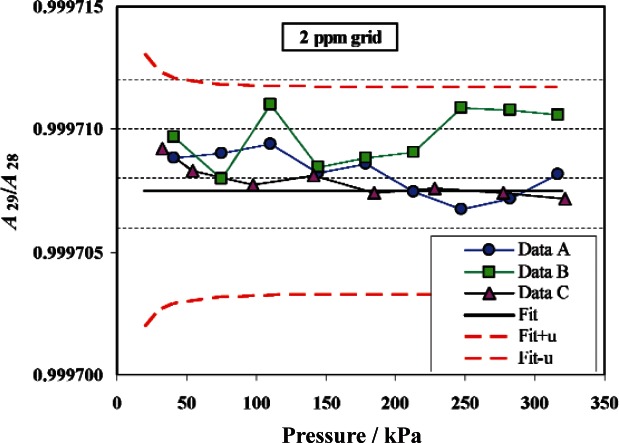
Area ratio of PG29 to PG28, A29/A28, from direct comparison (Data A, Data B, and Data C) and from the ratio of fitted areas of each to PG38 and PG39 (Fit). The dashed lines are the ratio of fitted areas plus or minus the standard uncertainty of PG28. Data A and B from 2005, Data C from 2002.

An example of a characterization, in this case for PG28, is shown in Fig. 5. Effective area data is shown at 10 pressures for PG28 calibrated against both PG38 and PG39 in gauge mode, using Eq. (5) to calculate *A_e,T_*. The data is fit to Eq. (3) with *b*_1_ constrained to zero.^6^ The horizontal line through the center of the data is the fitted effective area. The upper and lower curves are the effective area plus and minus the standard uncertainty, *u*(*A_e_*). The uncertainty analysis will be discussed in more detail in Sec. 4.4; in summary, the largest component of uncertainty for PG28 is the uncertainty in effective area of the primary standard, with the next largest component being the uncertainty in the masses used on the primary standard and on PG28. The relative standard uncertainty is lowest at the highest pressure, with a value of 4.2 × 10^−6^ (4.2 ppm). The comparison of the area ratio between PG29 and PG28 (the two TTL gauges) is shown in Fig. 6. The solid line in the center is the ratio of fits of each gauge from the primary standards. The data points are comparative calibrations of PG29 against PG28. Data sets A, B, and C used different combinations of masses, different columns for mounting the gauges, or calibrations separated in time. The uncertainty bands are the relative standard uncertainty of the effective area of PG28. There is good agreement of the area ratio via the fit ratios and direct calibration.

**Fig. 7:**
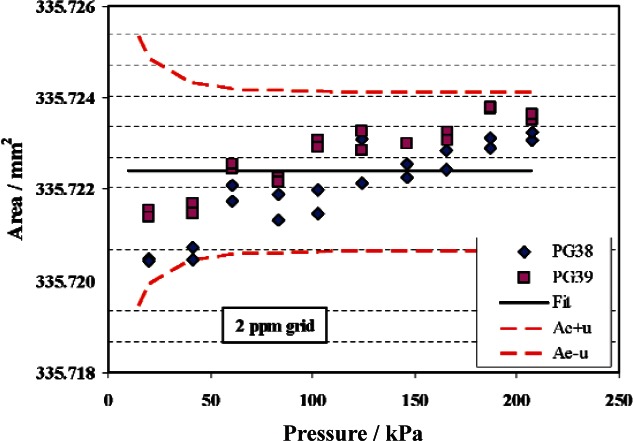
Effective area of PG22. Symbols are measured data from PG38 and PG39, solid line is fit of data with *b*_1_ constrained to zero, and dashed lines are the fitted area plus or minus the standard uncertainty.

The data and fit for TL gauge PG22 is shown in Fig. 7. As for the TTL gauge, the fit for PG22 constrains *b*_1_ to zero. Although there is some apparent positive slope in the area as a function of pressure, small force errors at low pressure could account for this slope, rather than distortion of the piston gauge. The possible decrease in area at low pressure is accounted for in the uncertainty analysis.

**Fig. 8:**
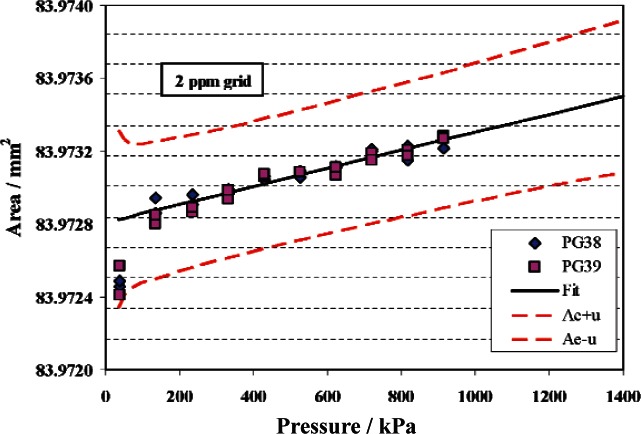
Effective area of PG34. Symbols are measured data from PG38 and PG39, solid line is fit of data, and dashed lines are the fitted area plus or minus the standard uncertainty..

The C series piston gauges are characterized with a minor modification from the method used for the TTL and TL series gauges. These gauges have a nominal diameter of 10.3 mm and are used up to 1.4 MPa, which requires extrapolation beyond the 914 kPa calibration pressure against the primary standards. Both secondary standards of the C series (PG34 and PG37) are fit with a *b*_1_ determined from the method of least squares. The data, fit, and standard uncertainty for PG34 are shown in Fig. 8.

### 4.2 Characterization of V Series Secondary Standards, 7 MPa Full Scale

The V series gauges (PG13 and PG35) are used up to 7 MPa and have a nominal diameter of 3.27 mm. These gauges are calibrated against the C series gauges rather than the primary standards. The area ratio of a V series to a C series gauge is 1:10 and the pressure overlap is only 20 % of full scale on the V series gauge. Extrapolating distortion coefficients obtained from fits of the V to C calibration data would produce unreasonably high uncertainties for the V series gauges at the higher operating pressures.

**Fig. 9:**
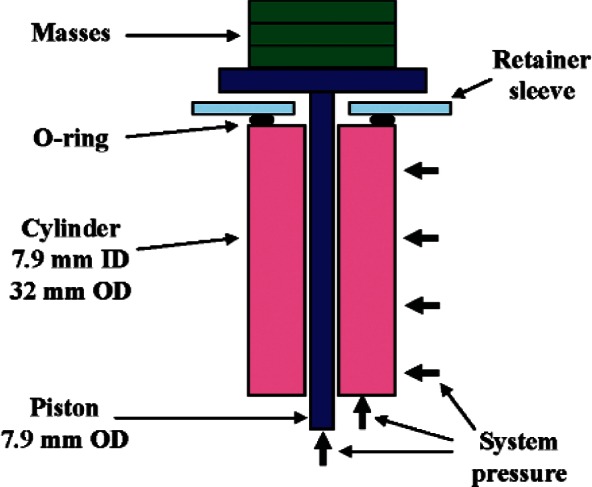
Schematic of PG7601 piston-cylinder module with 7.9 mm diameter piston. Cylinder inner diameter is 7.9 mm, outer diameter is 32 mm. O-ring provides pressure seal on top horizontal surface of cylinder. Lower surface and outer diameter of cylinder loaded at system pressure.

To determine the distortion coefficient of the V series gauges, we perform a calibration against an auxiliary piston gauge. This gauge is a DH Instruments PG7601 series gauge operated to 7 MPa. It has a geometry which lends itself to calculation of its distortion coefficient using elasticity theory (see Fig. 9). PG7601 has a uniform diameter piston (7.9 mm) and a uniform diameter cylinder (32 mm outer diameter); the seal on the cylinder is on the top surface so the cylinder is loaded radially inward and vertically upward by the full system pressure. The upward force is balanced by the seal on the top surface of the cylinder. The calculated distortion coefficient is:

(6) $$b_{1,7601}=-2.360\times 10^{-12}{\text{Pa}}^{-1}.$$

One of the V series gauges, PG13, was calibrated against PG7601 from 700 kPa to 7 MPa. The measured distortion difference was 5.021 × 10^−12^ Pa^−1^. The distortion of PG13 is determined from:

(7) $$\begin{array}{l} b_{1,PG13}=\left(b_{1,PG13}-b_{1,7601}\right)+b_{1,7601}\\ =5.021\times 10^{-12}{\text{Pa}}^{-1}-2.360\times 10^{-12}{\text{Pa}}^{-1}\\ b_{1,PG13}=2.661\times 10^{-12}{\text{Pa}}^{-1}. \end{array}$$

**Fig. 10:**
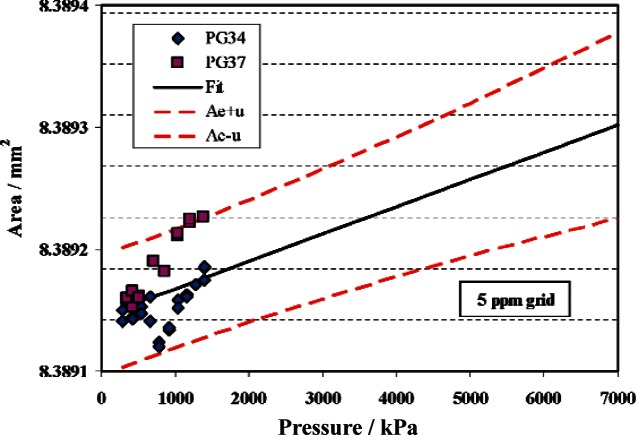
Effective area of PG13. Symbols are measured data from PG34 and PG37, solid line is fit of data with *b*_1_ constrained at 2.661 × 10^−12^ Pa^−1^, and dashed lines are the fitted area plus or minus the standard uncertainty.

The data from PG13 calibrated against the C series gauges is fit to Eq. (3) with *b*1,PG13 fixed at the value in Eq. (7). Figure 10 shows the result. The figure emphasizes the limited overlap in pressure between the C and V series gauges. The relative standard uncertainty ranges from 5.8 × 10^−6^ to 9.0 × 10^−6^, with the uncertainty increasing as the pressure increases from the mean pressure of the calibration data (830 kPa).

### 4.3 Characterization of D Series Secondary Standards, 17 MPa Full Scale

The D series gauges (PG23 and PG32) are used up to 17 MPa and have a nominal diameter of 3.27 mm. They are characterized by calibration against the V series gauges up to the common overlap pressure of 6.9 MPa, which is 41 % of the D series full scale pressure. Distortion in the D series gauges is significant, and extrapolation is required beyond 6.9 MPa.

When PG23 and PG32 are each calibrated up to 6.9 MPa against the V series gauges, the fitted *b*_1_ coefficients are −5.9 × 10^−13^ Pa and −10.1 × 10^−13^ Pa, respectively. A direct calibration of PG23 against PG32 up to 17 MPa gives a fitted difference in *b*_1_ between the gauges of 1.4 × 10^−13^ Pa; the Type A standard uncertainty of this difference is larger than the fitted difference. In other words, direct calibration of PG23 against PG32 over the full pressure range gives the same distortion for both to within experimental uncertainty. For PG23 and PG32, we assign a value of *b*_1_ to both that is the average of the values independently determined by the calibrations against the V series gauges.

**Fig. 11:**
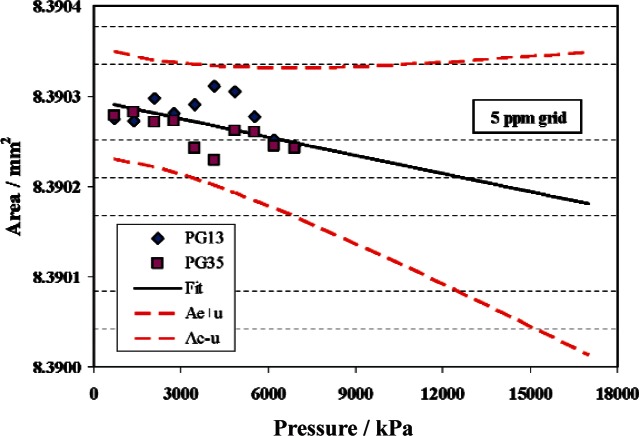
Effective area of PG23. Symbols are measured data from PG13 and PG35, solid line is fit of data with *b*_1_ constrained at −7.968 × 10^−13^ Pa^−1^, and dashed lines are the fitted area plus or minus the standard uncertainty.

The calibration data is then fit to Eq. (3) with *b*_1_ fixed. The results for PG23 are shown in Fig. 11. As with the V series gauges, the uncertainty grows with increasing pressure, due to the uncertainty in the distortion coefficient.

### 4.4 Uncertainties in Secondary Standard Piston Gauges

The uncertainty in effective area of the secondary standards is evaluated using methods described in [11]. Type A standard uncertainties are evaluated by statistical methods. Type B standard uncertainties are evaluated in two ways: (1) the law of propagation of uncertainty as applied to the measurement equation, Eq. (5); and (2) estimates of other effects. The uncertainty components are added in quadrature, and a coverage factor (*k* = 2) is multiplied by the standard uncertainty to give an expanded uncertainty. A more complete discussion of the uncertainties in the calibration of two piston gauges is given in [12].

The Type A uncertainty is given by the standard deviation of the predicted value of the fit. For the cases when *b*_1_ is either zero or fixed (but not determined through the fitting routine), this uncertainty is the standard error of the fit divided by *n*^1/2^, where *n* is the number of observations. This applies to the TL, TTL, V, and D series gauges. For the C series gauges, *b*_1_ is determined by the least squares fitting routine applied to Eq. (3). The general form of the Type A relative standard uncertainty in this case is:

(8) $$\frac{u_{A}(A_{e})}{A_{e}}={\left(\frac{\sigma_{\mathrm{fit}}^{2}}{A_{0}^{2}n}+{\left(\sigma_{b_{1}}\cdot (p-p_{m})\right)}^{2}\right)}^{1/2}.$$

Here, *σ_fit_* is the standard error of the fit, *σ_b_*_1_ is the standard error of coefficient *b*_1_, and *p_m_* is the mean pressure of the calibration data. Eq. (8) shows that the Type A uncertainty is a minimum at pressure *p_m_*, and increases as the pressure departs from that value.^7^ For all secondary standards, the Type A relative standard uncertainty is less than 1 × 10^−6^, and in most cases is less than 0.5 × 10^−6^. Typical values for *n* range from 20 to 40.

Type B uncertainties arising from the measurement equation are evaluated in the usual manner described in [11]. Partial derivatives are taken of *A_e,T_* with respect to the parameters on the right hand side of Eq. (5), the so-called sensitivity coefficients, and the standard uncertainty of the parameters are evaluated. The product of each sensitivity coefficient and the standard uncertainty is referred to as the uncertainty in effective area due to that parameter. Those values are added in quadrature, giving the variance, and the combined Type B standard uncertainty is the square root of the variance. Table 2 lists the Type B standard uncertainties for PG28 operated at 300 kPa. Within Table 2, the three left-most columns list the components and their nominal values at *p* = 300 kPa; columns 4 to 6 give the definition of the sensitivity coefficients and their values at 300 kPa; columns 7 and 8 list the standard uncertainty of the components; and the final column on the right lists the relative standard of that component on *A_e,T_*.

The largest relative standard uncertainty arising from the measurement equation is that due to the effective area of the reference piston gauge, either the primary standard for the TTL, TL, and C series, or the secondary standard against which the V and D series gauges are calibrated. The magnitude is 3 × 10^−6^ (3 ppm) or higher. Since the V series gauges are 1-step removed from the primary standard and the D series gauges are 2-steps removed, the uncertainty component due to the effective area of the reference gauge is successively larger. The next largest component on a relative basis is that due to the masses. Each calibration requires two mass sets, which are generally not interchangeable since the reference and test piston gauges are often of different diameters. NIST assumes the masses within a set are perfectly correlated, and the masses between sets are uncorrelated, which is the most conservative estimation for this uncertainty component. We use a relative standard uncertainty of each mass element of 2 × 10^−6^ (2 ppm), so the combination of the two sets is an uncertainty in effective area due to the masses of 2.8 × 10^−6^ (2.8 ppm). Relative uncertainties in effective area due to temperature uncertainties of the two piston gauges taken together are less than 1 × 10^−6^; relative uncertainties in effective area due to thermal expansion uncertainties of the two piston gauges are also less than 1 × 10^−6^. Relative uncertainties in effective area due to uncertainties in the density of the masses, local acceleration of gravity,^8^ density of the gas pressurizing the piston gauges, and height difference between the reference levels when taken together are usually less than 0.3 × 10^−6^.

There are several other uncertainty components that must be included for the secondary standards that are not part of the measurement equation, depending on the series. For the TTL, TL, and C series gauges a “low pressure” uncertainty component is included due to the possibility of force errors at low pressure. Data from the calibrations often show a departure from a straight line as the pressure approaches zero. To estimate this uncertainty, each data set is fit to an additional model equation given by:

(9) $$A_{e}=A_{0}(1+b_{1}p)-t/p,$$

with “tare” parameter *t* determined by the method of least squares. The function given by Eq. (9) is such that the *t*/*p* term becomes larger as *p* goes to zero, and the fit departs more from a straight line (for the TTL and TL gauges, *b*_1_ is fixed at zero). Eqs. (9) and (3) represent two possible models to the experimental data. The difference in fits is assumed to represent the half-width of a rectangular distribution of the possible true value of effective area; hence the uncertainty component due to ignoring the tare component is taken as $\frac{t/p}{\sqrt{3}}$. In Table 2, this component is denoted as “fit error, low pressure.”

Another uncertainty component results from the method of determining pressure equilibrium between the reference and test piston gauges. If a differential pressure cell is used to balance pressures, a standard uncertainty is taken as the smallest mass difference on one of the gauges that produces a measurable deflection in the cell.^9^ The low pressure and pressure equilibrium components taken together represent a relative uncertainty in PG28 of 3.7 × 10^−6^ at 20 kPa (the minimum pressure), and 3.4 × 10^−6^ at 35 kPa for PG34.

For the C series gauges, an uncertainty component due to extrapolation is included since the gauge is used up to 1.4 MPa, beyond the maximum pressure of calibration against the primary standard. The uncertainty of the extrapolation is evaluated from multiple calibrations of PG34 against the primary standards, rather than the fit uncertainty of *b*_1_ from a single calibration. Determination of a fitted *b*_1_ from an individual set of data can be unduly influenced by force errors due to mass errors or other low pressure effects that present themselves as systematic. PG34 was calibrated against PG39 multiple times with different combinations of mass sets on PG34, and *b*_1_ was determined for each calibration. Assuming the fitted distortion coefficients represent a rectangular distribution of possible values, the standard uncertainty *u*(*b*_1_) is taken as the half-width of the distribution divided by $\surd \bar{3}$. Although this component was determined for PG34 only, it is assumed to be the same for all C series gauges. When the gauge is operated at the average pressure given by the calibration data, this component is zero since the effective area at that pressure does not depend on *b*_1_. Hence this component grows linearly as the pressure departs from the average pressure. At 1.4 MPa, the relative standard uncertainty due to extrapolation is 2 × 10^−6^.

The V series gauges are calibrated against the C series gauges for effective area up to 1.4 MPa, and against PG7601 to determine the distortion coefficient. An additional Type B uncertainty component is included to estimate the uncertainty due to the distortion from 1.4 MPa to 7 MPa, the region of calibration against PG7601. We take as the standard uncertainty the same value used for PG38 and PG39, *u*(*b*_1_) = 1.12×10^−12^ Pa^−1^. As confirmation that *b*_1_ and *u*(*b*_1_) are reasonable, we look at several other methods for estimating *b*_1_ of PG13 that do not depend on the elasticity theory calculations of PG7601. In one method, Bowers and Olson [13] used a capacitive technique to measure the distortion of PG13. They estimated *b*_1_ of 1.88 × 10^−12^ Pa^−1^ for PG13. A second method used a finite element model and a gas flow model to calculate *b*_1_ of PG13. In a third method, *b*_1_ of PG13 was determined from the distortion of hydraulic primary standard PG27, calibration of PG23 against PG27, and calibration of PG13 against PG23. The range in *b*_1_ from these alternate methods is 1.09 × 10^−12^ to 3.30 × 10^−12^ Pa^−1^; the difference in *b*_1_ for PG13 from the chosen method and these alternatives is − 0.64 × 10^−12^ to +1.57 × 10^−12^ Pa^−1^. Hence *u*(*b*_1_) is consistent with differences in *b*_1_ from alternatives to the chosen method. At 7 MPa, the relative standard uncertainty in effective area due to extrapolation is 7 × 10^−6^.

For the D series gauges, the calibration range against the V series extends up to 6.9 MPa, which is 40 % of the full scale pressure. The Type B distortion uncertainty from the V series is used for the full range of the D series. The Type A uncertainty in the fitted distortion coefficient (resulting from the comparison of PG23 to PG32) is added in quadrature, although it is small compared to the uncertainty from the V series extrapolation. At 17 MPa, the relative standard uncertainty due to the uncertainty in distortion is 19 × 10^−6^.

As a final uncertainty component, we consider the effect of using the secondary standard gauges in absolute mode, even though the comparisons to the primary standards and each other were performed in gauge mode. We have found that the effective area of PG28, when calibrated against the UIM, differs by as much as *a* = 2 × 10^−6^ on a relative basis when operated in gauge vs. absolute mode. We assume all secondary standard gauges could exhibit a difference in gauge and absolute mode, although we use the same effective area for both modes. When used in absolute mode, we add a relative standard uncertainty of 2 × 10^−6^ in quadrature; statistically this means that there is a 2 out of 3 chance that effective area in absolute mode, *A_ea_*, lies in the interval from *A_e_* − *a* to *A_e_*+ *a*.

### 4.5 Combined Standard Uncertainties for Secondary Standard Piston Gauges

Examination of the uncertainty components for the TL, TTL, C, D, and V series gauges shows that various components can be grouped together to form a general uncertainty equation with parameter values that depend on the particular gauge used. Components on a relative basis are either constant with pressure, inversely proportional to pressure, are linearly proportional to pressure. The uncertainty is calculated from:

(10) $$\frac{u(A_{e})}{A_{e}}={\left[{\left(\frac{c_{1}}{p}\right)}^{2}+c_{2}^{2}+{\left(c_{3}\cdot (p-p_{\mathrm{ave}})\right)}^{2}+{\left(c_{4}\cdot p\right)}^{2}\right]}^{1/2}.$$

**Fig. 12:**
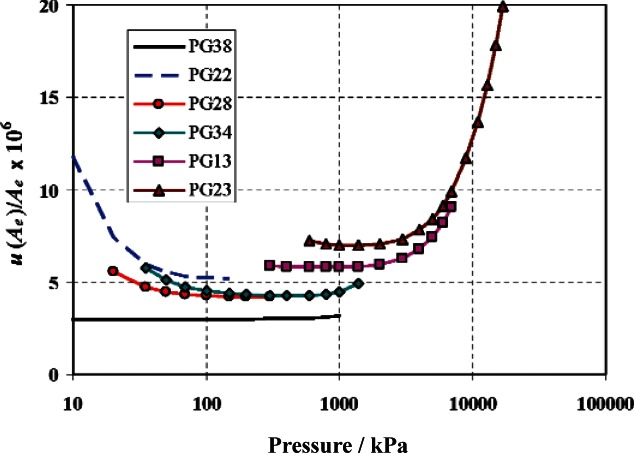
Operating ranges and relative standard uncertainties of NIST gas piston gauges.

Parameters *c*_1_, *c*_2_, *c*_3_, *c*_4_, and *p_ave_* are unique for each gauge. Table 3 lists the values of the parameters for all the primary and secondary standard gauges for gauge mode. Standard uncertainties for one gauge of each series are plotted in Fig. 12, along with the uncertainty of the primary standard PG38. As can be seen, uncertainties are lowest in the region of overlap with the primary standard. At low pressure, uncertainties increase due to force errors; at high pressure, uncertainties increase due to the uncertainty in the distortion coefficients.

When a piston gauge is used to generate pressure, the standard uncertainty in pressure is larger than the uncertainty in effective area. As can be seen through Eq. (4), additional uncertainties due to mass, density, gravity, thermal expansion, and temperature must be included. For typical uses, the combined relative standard uncertainty of these components is approximately 2.5 × 10^−6^, which is added in quadrature to the uncertainty in effective area.

## 5. Validation of Uncertainties Through International Comparisons

One method for independently validating the pressures realized by the piston gauges and their stated uncertainties is to compare the NIST standards to those of other National Metrology Institutes (NMIs). The formal process for this is described in the Mutual Recognition Arrangement (MRA) of the Comité International des Poids et Mesures (CIPM) [14]. Comparisons between the standards of NMIs administered through the CIPM are designated is Key Comparisons. Key Comparisons provide the agreement (or equivalence) of the standards to a Key Comparison Reference Value (KCRV). They also provide results on the agreement between the standards of the NMIs.

Key Comparison CCM.P-K1c compared the standards of 5 NMIs from 79 kPa to 6.79 MPa during the time period from 1998 to 1999 [15]. NIST was the co-pilot of the comparison, which used two NIST piston gauge artifacts as the transfer standards. The NMIs determined the effective area of the transfer standards by measurements against their pressure standards. The measurand for determining equivalence was the effective area of the transfer standards. NIST re-measured the effective area of the two transfer standards using the new values of the gas pressure scale, and compared those values to the KCRV and other participant values determined in CCM.P-K1c.^10^

**Fig. 13:**
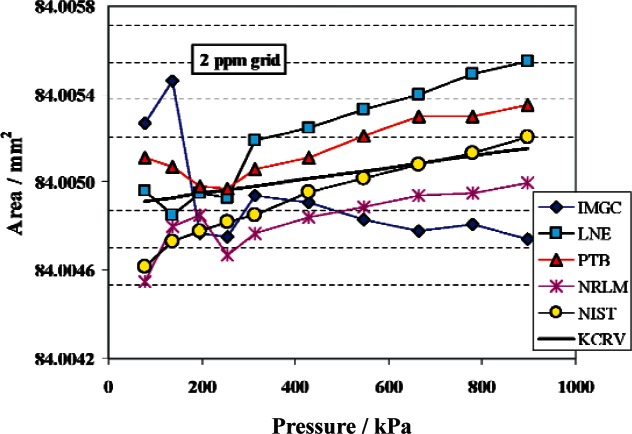
Effective area of piston gauge C-415 as measured by IMGC, LNE, PTB, and NRLM as part of CCM.P-K1c, KCRV from CCM.P-K1c, and re-measured by NIST traceable to primary standards PG38 and PG39.

Over the range of 79 kPa to 896 kPa, the transfer standard was C series piston gauge C-415. NIST re-measured effective areas of C-415 by calibration against PG38 and PG39. The effective areas measured by NIST at the 10 pressure points are listed in Table 4, along with the KCRV, the difference, *D*, between the NIST value and the KCRV, and the standard uncertainties. The degree of equivalence is given by the pair of numbers, *D* and *u*(*D*). If *D* / *u*(*D*) is less than 1 there is equivalence at the *k* = 1 level; if *D*/(2*u*(*D*)) is less than 1 there is equivalence at the *k* = 2 level. The reference temperature for the NIST value was taken as 20 °C for consistency with the Key Comparison. Table 4 shows that there is full equivalence to the reference value at *k* = 1. Figure 13 shows the NIST values and the results from the other NMIs of the comparison. Although the uncertainties are not shown, NIST is equivalent to the 4 other NMIs at *k* = 1 for all pressures.

**Fig. 14:**
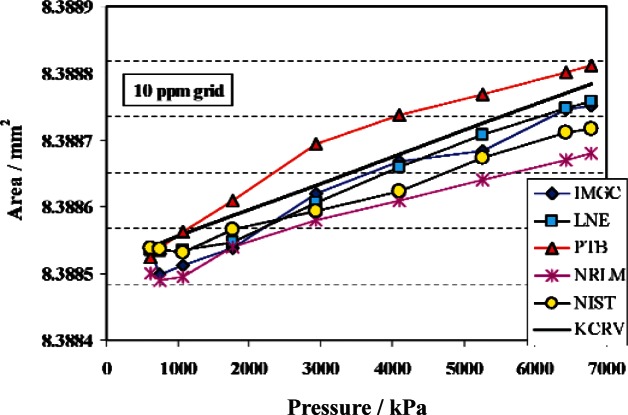
Effective area of piston gauge V-762 as measured by IMGC, LNE, PTB, and NRLM as part of CCM.P-K1c, KCRV from CCM.P-K1c, and re-measured by NIST traceable to primary standards PG38 and PG39.

A second artifact in CCM.P-K1c compared effective areas from 621 kPa to 6789 kPa. This artifact was PG35 that is part of the NIST gas pressure scale. The results for NIST with PG35 re-measured against the C series piston gauges with their new traceability, along with the KCRV, are listed in Table 5. NIST is again fully equivalent to the reference value at *k* = 1. Figure 14 shows the NIST values and those of the other NMIs at the 9 pressures of the comparison. NIST is equivalent at the *k* = 1 level at all pressures when compared to IMGC, LNE, and NRLM. For PTB, NIST is equivalent at *k* = 1 for 7 of the 9 pressures, and equivalent at *k* = 2 for all 9 pressures.

A similar comparison of the new gas pressure scale to NPLI using an artifact owned by them showed full equivalence at *k* = 1 from 0.4 to 4.0 MPa [16].

## 6. Conclusions

NIST’s gas pressure scale is now based on primary standard piston gauges and secondary standard piston gauges which are traceable to those primary standards. The reduced uncertainties made possible by this pressure scale were formally adopted in September 2008 by the BIPM by acceptance of NIST’s declared Calibration Measurement Capabilities (CMCs). The foundation of the scale is two 35.8 mm diameter piston gauges which have been dimensionally characterized and carefully evaluated against each other to determine their effective area and distortion with pressure. These piston gauges (PG38 and PG39) have expanded relative uncertainties that approach those of NIST’s ultrasonic interferometer manometer. Comparing the secondary standard piston gauges to PG38 and PG39 instead of a mercury manometer offers two advantages that contribute to the low uncertainties. The first is that the primary standards operate to 1 MPa, allowing direct comparison of the TL, TTL, and C series gauges. This eliminates most of the uncertainty due to extrapolation which was required in the old scale. The second advantage is that piston gauges are easier to use than manometers, making it feasible to repeat comparisons more often.

A piston gauge generates pressure by balancing gravitational forces of traceable masses with hydraulic pressure acting over an “effective area.” At a National Metrology Institute such as NIST, a piston gauge is most often used to calibrate a customer’s piston gauge, which the customer then uses to generate pressure or perhaps calibrate other piston gauges. Hence the traceability chain involves transferring the effective area rather than pressure. The uncertainty analysis of the calibration of piston gauges against each other will be similar to that summarized in Table 2. If temperatures are kept close to the reference temperature and the piston diameters are of similar size, most of the increase in uncertainty of the calibrated gauge will be due to the mass uncertainty and the Type A uncertainty of the calibration. Techniques such as mass switching between the NIST and customer piston gauges can minimize the uncertainty due to mass.

NIST can now provide uncertainties to calibration customers that are pressure dependent for each secondary standard used in the calibration. Over a wide range of pressure (20 kPa to 7 MPa), the relative standard uncertainty of the NIST piston gauges is less than 10 × 10^−6^, and over a more limited range (30 kPa to 1.4 MPa) it is less than 5 × 10^−6^.

## Figures and Tables

**Table 1:** Characteristics of piston gauges used in NIST gas pressure scale. Diameters given are nominal. Effective area coefficients are valid for the entire range in pressure and are defined in Eq. (3). For PG38 and PG39, *b*_1_ is given for the upright configuration

Piston Gauge		Diameter	Effective area coefficients		Range in *p* / kPa	
Name	Series	/mm	*A*_0_ / m^2^	*b*_1_ / Pa^−1^	Low	High
PG38	N / A	35.8	1.0079497E-03	8.97E-12	20	1000
PG39	N / A	35.8	1.0079484E-03	8.97E-12	20	1000
PG22	TL	20.7	3.357224E-04	0	10	150
PG36	TL	20.7	3.357388E-04	0	10	150
PG28	TTL	20.7	3.358209E-04	0	20	300
PG29	TTL	20.7	3.357227E-04	0	20	300
PG34	C	10.3	8.397281E-05	5.903E-12	35	1400
PG37	C	10.3	8.398156E-05	8.319E-12	35	1400
PG13	V	3.27	8.398145E-06	2.661E-12	360	7000
PG35	V	3.27	8.388724E-06	4.267E-12	360	7000
PG23	D	3.27	8.390295E-06	−7.968E-13	700	17000
PG32	D	3.27	8.389404E-06	−7.968E-13	700	17000

**Table 2:** Type B uncertainty contributions to combined standard uncertainty of PG28 at 300 kPa. Largest components are uncertainty in area of primary standard and uncertainties in mass on PG28 and primary standard. Type A relative standard uncertainty is 1.53 × 10^−7^

Type B uncertainty component			Sensitivity coefficient divided by *A_e,T_*			Uncertainty (*k* = 1)		Rel. unc. of term on *A_e,T_*
Term	Value	Units	Definition	Abs. Value	Units	Value	Units	
*A e,_R_*	1.01E-03	m^2^	1/*A_e,R_*	9.92E+02	M^−2^	3.04E–09	m^2^	3.02E–06
*M_R_*	30.9	kg	−1/*M_R_*	3.24E–02	Kg^−1^	6.17E–05	kg	2.00E–06
*M_T_*	10.3	kg	1/*M_T_*	9.73E–02	Kg^−1^	2.06E–05	kg	2.00E–06
*ρ_a_*	1.18	kg / m^3^	Δ*ρ_M_*/(*ρ_M,T_ρ_M,R_*)−*gh*/*p_T_*	9.51E–06	m^3^ / kg	0.010	kg / m^3^	9.51E–08
*ρ_M,R_*	7840	kg / m^3^	Δ*ρ_a_*/*ρ_M,R_*^2^	1.63E–10	m^3^ / kg	45.3	kg / m^3^	7.36E–09
*ρ_M,T_*	7800	kg / m^3^	−Δ*ρ_a_*/*ρ_M,T_*^2^	1.64E–10	m^3^ / kg	45.0	kg / m^3^	7.40E–09
*g*	9.80	m / s^2^	(*ρ_f_* – *ρ_a_*)*h* / *p_T_*	3.62E–06	s^2^ / m	9.80E–06	m / s^2^	3.55E–11
*α_p,R_* + *α_c,R_*	8.75E-06	K^−1^	*T_R_* – 23.00	0.50	C	3.00E-08	K^−1^	1.50E–08
*α_p,T_* + *α_c,T_*	9.10E-06	K^−1^	*–*(*T_T_*–23.00)	0.50	C	5.25E-07	K^−1^	2.63E–07
*T_R_*	22.5	C	*α_p,R_+ α_c,R_*	8.75E-06	K^−1^	0.058	C	0.05E–07
*T_T_*	22.5	C	−(*α_p,T_ + α_c,T_*)	9.10E–06	K^−1^	0.058	C	5.25E–07
*ρ_f_*	4.67	kg / m^3^	*gh* / *p_T_*	1.02E–05	m^3^ / kg	0.0047	kg / m^3^	4.75E–08
*h*	−0.311	m	${\left(\rho_{f}-\rho_{a}\right)}_{g/p_{T}}$	1.14E–04	1 / m	0.002	m	2.28E–07
Pressure equilibrium	0.049	Pa	1/*p_T_*	3.33E–06	1 / Pa	0.049	Pa	1.62E–07
Fit error, low pressure	0.046	Pa	1/*p_T_*	3.33E–06	1 / Pa	0.046	Pa	1.52E–07
						**Sum of all terms**		**4.22E-06**

Notes:

1. Δ*ρ_M_=ρ_M,R_ – ρ_M,T_*

2. Δ*ρ_a_* is the difference in air density between that measured during the calibration of the masses and the use of the masses on the piston gauge. Its value is taken as 0.01 kg/m^3.^

**Table 3:** Coefficients used in calculating the relative standard uncertainty in effective area of gas piston gauges, given by $\frac{u(A_{e})}{A_{e}}{\left[{\left(\frac{c_{1}}{p}\right)}^{2}+c_{2}^{2}+{\left(c_{3}\cdot (p-p_{\mathrm{ave}})\right)}^{2}+{\left(c_{4}\cdot p\right)}^{2}\right]}^{1/2}$ · *p* is in Pa. Lowest and highest relative standard (*k* = 1) uncertainty over operating pressure range also shown. Uncertainties for PG38 and PG39 valid for gauge and absolute mode. For other piston gauges, coefficients given for gauge mode; for absolute more, add 2.0 × 10^−6^ in quadrature with coefficient *c*_2_

Piston Gauge	Coefficients for *u* / *A_e_*, *k* = 1					Range in *u* / *A_e_* × 10^6^	
	*c* 1 / Pa	*c*_2_	*c*_3_ / Pa^−1^	*p_ave_* / Pa	*c*_4_ / Pa^−1^	low	high
PG38	0	3.00E-06	0	0	1.12E-12	3.0	3.2
PG39	0	3.00E-06	0	0	1.12E-12	3.0	3.2
PG22	0.106	5.11E-06	0	0	1.12E-12	5.2	11.8
PG36	0.109	5.11E-06	0	0	1.12E-12	5.2	12.0
PG28	0.073	4.21E-06	0	0	1.12E-12	4.2	5.6
PG29	0.147	4.22E-06	0	0	1.12E-12	4.3	8.5
PG34	0.133	4.20E-06	2.33E-12	520335	1.12E-12	4.3	5.8
PG37	0.144	4.21E-06	2.36E-12	530847	1.12E-12	4.3	6.0
PG13	0.167	5.82E-06	1.12E-12	828704	0	5.8	9.0
PG35	1.180	6.43E-06	1.14E-12	828704	0	6.5	9.5
PG23	1.349	6.87E-06	1.16E-12	828704	0	7.0	20.0
PG32	1.349	6.89E-06	1.16E-12	828704	0	7.0	20.0

**Table 4:** Effective area of C-415 measured by NIST, the KCRV from CCM.P-K1c, the difference (*D*) between the NIST value and the KCRV, and the associated uncertainties. Reference temperature for the NIST value is 20 °C for consistency with CCM.P-K1c

	New NIST Value			KCRV			Degree of Equivalence		
*P* / kPa	*A_e_* / mm^2^	*u* (*A_e_*) / mm^2^	*u* (*A_e_*) / *A_e_* (× 10^6^)	*A_e_* / mm^2^	*u* (*A_e_*) / mm^2^	*u* (*A_e_*) / *A_e_* (× 10^6^)	*D* / *A_e_* (× 10^6^)	*u* (*D*) / *A_e_* (× 10^6^)	*D* / *u* (*D*)
79.4	84.00462	0.000389	4.63	84.00491	0.00021	2.50	−3.53	5.26	−0.67
137.8	84.00473	0.000378	4.50	84.00493	0.00021	2.50	−2.37	5.15	−0.46
196.0	84.00478	0.000374	4.45	84.00495	0.00021	2.50	−2.03	5.10	−0.40
254.5	84.00482	0.000373	4.43	84.00497	0.00021	2.50	−1.75	5.09	−0.34
312.8	84.00485	0.000374	4.45	84.00498	0.00021	2.50	−1.57	5.10	−0.31
429.5	84.00495	0.000373	4.44	84.00502	0.00021	2.50	−0.75	5.09	−0.15
546.2	84.00502	0.000374	4.45	84.00505	0.00021	2.50	−0.40	5.10	−0.08
663.0	84.00508	0.000375	4.46	84.00509	0.00021	2.50	−0.07	5.12	−0.01
779.7	84.00513	0.000377	4.49	84.00512	0.00021	2.50	0.14	5.14	0.03
896.4	84.00521	0.000380	4.52	84.00516	0.00021	2.50	0.60	5.17	0.12

**Table 5:** Effective area of V-762 (PG35) measured by NIST, the KCRV from CCM.P-K1c, the difference (*D*) between the NIST value and the KCRV, and the associated uncertainties. Reference temperature for the NIST value is 20 °C for consistency with CCM.P-K1c

	New NIST Value			KCRV			Degree of Equivalence		
*P* / kPa	*A_e_* / mm^2^	*u* (*A_e_*) / mm^2^	*u* (*A_e_*) / *A_e_* (× 10^6^)	*A_e_* / mm^2^	*u* (*A_e_*) / mm^2^	*u* (*A_e_*) / *A_e_* (× 10^6^)	*D* / *A_e_* (× 10^6^)	*u* (*D*) / *A_e_* (× 10^6^)	*D* / *u* (*D*)
621	8.388538	5.80E-05	6.92	8.388541	6.04E-05	7.20	−0.32	9.99	−0.03
738	8.388537	5.75E-05	6.85	8.388546	6.04E-05	7.20	−1.09	9.94	−0.11
1077	8.388531	5.70E-05	6.79	8.388559	6.04E-05	7.20	−3.31	9.90	−0.33
1766	8.388566	5.62E-05	6.70	8.388586	6.04E-05	7.20	−2.46	9.84	−0.25
2934	8.388594	5.77E-05	6.88	8.388632	6.04E-05	7.20	−4.61	9.96	−0.46
4103	8.388622	6.15E-05	7.33	8.388679	6.04E-05	7.20	−6.71	10.28	−0.65
5271	8.388673	6.81E-05	8.12	8.388725	6.04E-05	7.20	−6.12	10.85	−0.56
6439	8.388711	7.57E-05	9.02	8.388771	6.04E-05	7.20	−7.14	11.54	−0.62
6789	8.388717	7.50E-05	9.36	8.388785	6.04E-05	7.20	−8.06	11.81	−0.68

## Footnotes

1 For piston gauges operating liquids, surface tension forces are significant and must be added to the gravitational forces.

2 NIST uses a gas piston gauge up to 100 MPa that is calibrated against a hydraulic piston gauge. It is not included in the present paper.

3 In “absolute mode,” the pressure surrounding the gauge and masses is zero. In “gauge mode,” atmospheric pressure surrounds the gauge and masses.

4 Certain commercial equipment, instruments, or materials are identified in this paper to foster understanding. Such identification does not imply recommendation or endorsement by NIST, nor does it imply that the materials or equipment identified are necessarily the best available for the purpose.

5 Throughout this document, lower-case *u* variables refer to standard (coverage factor, *k* = 1) uncertainties; upper-case *U* variables refer to expanded (*k* = 2) uncertainties.

6 *b*_1_ is constrained to zero because its fitted value using least squares is on the same order as its uncertainty, indicating the data can be described adequately using a lower order model.

7 Because of the coupling between *A*_0_ and *b*_1_ in Eq. (3), there are non-zero covariance terms in *u_A_*(*A_e_*) that have not been listed in Eq. (8). However, these terms are insignificant compared to variances from *A*_0_ and *b*_1_.

8 The same standard uncertainty in gravity will have a much larger relative effect on the uncertainty in pressure (Eq. 4) than the uncertainty in effective area (Eq. 5). In an effective area characterization, both mass sets are subjected to the same gravitational acceleration, so its uncertainty only affects the correction due to reference levels.

9 It is likely there is some correlation between the pressure equilibrium and the Type A uncertainty from the fitted equation. However, we have included both so as to not underestimate the uncertainty.

10 Although the NIST values for the effective area of the transfer standards were used to define the KCRV in CCM.P K1c, the KCRV was not re-calculated using the recent measurements of NIST.
